# The Beat

**DOI:** 10.1289/ehp.120-a348b

**Published:** 2012-08-31

**Authors:** Erin E. Dooley

## Smoking Policies Tighten in Halls of Academe

As the fall 2012 semester gets under way, at least 744 U.S. colleges and universities have made their campuses completely smoke free, indoors and out, and nearly three-quarters of those have banned all forms of tobacco on campus.^^1^^ Ty Patterson, executive director of the National Center for Tobacco Policy, told the *Christian Science Monitor*^^2^^ that many colleges were prompted to make the change by the U.S. Surgeon General’s 2006 statement that secondhand smoke is hazardous at any exposure level. A 2011 survey of nearly 28,000 college students at 44 schools reported daily smoking among 4.6% and occasional smoking in the past 30 days among 9.7%.^^3^^

## China Bans Shark Fins for Official Fetes

China’s Government Offices Administration of the State Council has announced it will issue guidelines to ban serving shark fin, a traditional delicacy, at official receptions.^^4^^ Final guidelines are expected within one to three years. In addition to helping conserve shark populations around the world, the ban may also help limit human consumption of β-methylamino-l-alanine (BMAA), a cyanobacterial neurotoxin found in high concentrations in shark, as well as in other contaminated seafood and shellfish, drinking water supplies, and recreational waters.^^5^^ Shark meat also typically contains high levels of methylmercury, and anecdotal reports suggest fins may be treated with formaldehyde before they are sold.^^5^^

**Figure:**
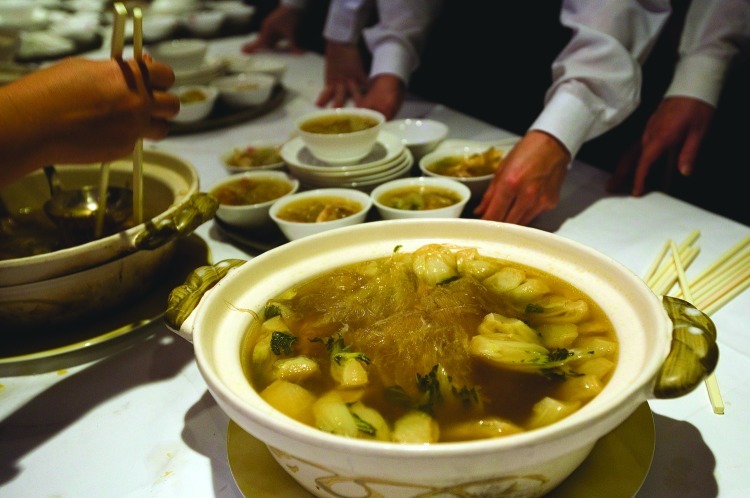
The Chinese government will no longer serve shark-fin soup at official receptions. © 2012 Paul Hilton/EPA/Corbis

## New C8 Panel Findings

For several decades DuPont’s Washington Works Plant in Parkersburg, West Virginia, released perfluorooctanoic acid (C8) into local waters, where it made its way into drinking water. The C8 Science Panel, appointed by the Wood County Circuit Court as part of a class-action legal settlement with DuPont, now reports finding probable links between exposure to C8 and ulcerative colitis and thyroid disease.^^6^^ In earlier reports the panel identified similar C8 links to cancers of the kidney and testicle and to pregnancy-induced hypertension. The panel has found no C8 link to several other diseases studied.

**Figure:**
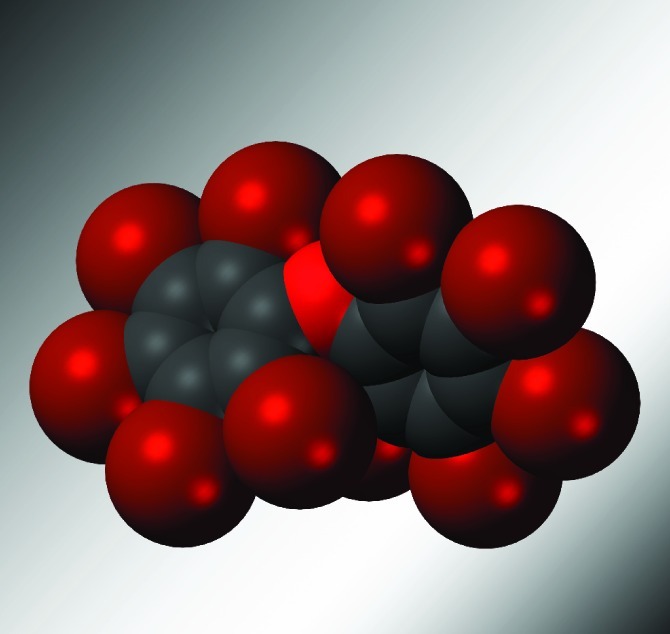
DecaBDE will be phased out of production by December 2013. Jynto

## EPA Solicits Comments on DecaBDE Substitute Report

Exposure to the persistent, bioaccumulative flame retardant decabromodiphenyl ether (decaBDE) has been linked to developmental health effects, and the compound is scheduled to be phased out of production by December 2013. In July 2012 the U.S. EPA Design for the Environment program released a comprehensive draft report on potential alternatives to the compound.^^7^^ The report will allow manufacturers to weigh the pros and cons of 30 alternatives, all of which are already on the market. Public comments on the report are being accepted through the end of September 2012.

## More Scientists Call for an End to Asbestos

Asbestos use is largely banned in industrialized nations because of health concerns, but imports are growing in poorer nations. In July 2012 the Joint Policy Committee of the Societies of Epidemiology, representing 12 member organizations, issued a joint statement calling for exporting nations to cease production of all forms of asbestos and for importing nations to cease its use.^^8^^ The statement follows the June 2012 approval of a $58-million loan by the Quebec provincial government to reopen and expand the Jeffrey mine, Canada’s last remaining asbestos mine.^^9^^
